# Appendicitis up to Date

**Published:** 1905-03-04

**Authors:** 


### Appendicitis Up to Date.

It was very apparent, during the discussion which took place at a meeting of the Royal Medical and Chirurgical Society of London on Tuesday, that the last word has yet to be said on the treatment of inflammatory affections of the appendix. Notwithstanding the immense volume of literature on the subject which has been poured out in recent years, appendicitis still offers a fruitful field for scientific debate. The discussion to which we refer had been arranged so as to include the subsequent course and later history of cases of appendicitis after operation, and it was opened by Sir Frederick Treves, who was able to bring forward extensive statistics drawn both from private practice and from the records of the London Hospital. An imposing number of cases also had been collected and analysed by Mr. Symonds and others, who followed Sir Frederick Treves on the platform. Although the statistics quoted probably underestimate the frequency of post-operative complications and recurrences, because they mostly cover operations done in recent years only, Sir Frederick Treves found that among 230 patients operated upon for appendicitis, at a time when the acute manifestations had subsided, no fewer than 11 subsequently complained that they were not at all relieved by the operation. Of every hundred patients in whom an abscess in the appendix region is opened and drained, no fewer than seventeen per cent. afterwards suffer from recurrence or other more or less serious trouble. Failures may be due to a variety of causes. The attacks of pain which first brought the patient under the surgeon's care may not have had their origin in the appendix, and consequently removal of that organ has not brought relief. Conditions which have led to this mistake are renal calculus, movable kidney, inflammatory conditions of the gall-bladder, ovary, and other parts. Further explanations of recurrent attacks after operation are failure to remove the appendix, chronic sinuses due to a retained concretion, chronic colitis, adhesions, and so forth. Without dealing further with these causes of disappointment, or with the complications which may follow operation on the appendix such as thrombosis, or pulmonary complications, or ventral hernia, we should like to point out that they are sufficiently numerous and frequent to call for the earnest consideration and study of operating surgeons. Now that appendicitis is such a fashionable illness, it is quite probable that some degree of operative intemperance exists, and if this be so, there could be no better antidote than a study of those operations which have not proved beneficial to the patients.

We may take it for granted that the eminent physicians and surgeons who took part in the discussion on Tuesday, in expressing their views, reflected, more or less accurately, the trend of the general surgical opinion of to-day; it is important to note, therefore, that there was a general agreement amongst them as to the undesirability of delay in resorting to the knife where the diagnosis is clear. No one spoke in defence of leaving the diseased appendix unmolested, and no one appeared to regard that organ as being of the least use to the human organism. Sir William Macewen lately upheld the contrary view in which he is supported by one of the leading authorities on developmental morphology, who says that, so far from being a retrogressive vestigial relic, the appendix is an organ which, although present in the anthropoids, attains its highest degree of development in the human species, and therefore may be presumed to have some definite function to perform in mankind. As is natural, the physicians who had been invited to join in Tuesday's debate had not a great deal to say, for the subject under consideration was chiefly a surgical one; but we should like to see the physicians have an opportunity on some future occasion of discussing the prevention of appendicitis, for, after all, this is, or ought to be, the ideal attainment towards which medical men should strive, and an authoritative lead along these lines would be certainly of professional and ultimately of great public utility.

